# Understanding nurses’ and physicians’ fear of repercussions for reporting errors: clinician characteristics, organization demographics, or leadership factors?

**DOI:** 10.1186/s12913-015-0987-9

**Published:** 2015-08-14

**Authors:** Evan S. Castel, Liane R. Ginsburg, Shahram Zaheer, Hala Tamim

**Affiliations:** 1 Department of Geography and Planning / Dalla Lana School of Public Health, University of Toronto, Toronto, Canada; 2 Health Policy & Management, York University, Toronto, Canada; 3 School of Kinesiology & Health Science, York University, Toronto, Canada

## Abstract

**Background:**

Identifying and understanding factors influencing fear of repercussions for reporting and discussing medical errors in nurses and physicians remains an important area of inquiry. Work is needed to disentangle the role of clinician characteristics from those of the organization-level and unit-level safety environments in which these clinicians work and learn, as well as probing the differing reporting behaviours of nurses and physicians. This study examines the influence of clinician demographics (age, gender, and tenure), organization demographics (teaching status, location of care, and province) and leadership factors (organization and unit leadership support for safety) on fear of repercussions, and does so for nurses and physicians separately.

**Methods:**

A cross-sectional analysis of 2319 nurse and 386 physician responders from three Canadian provinces to the Modified Stanford patient safety climate survey (MSI-06). Data were analyzed using exploratory factor analysis, multiple linear regression, and hierarchical linear regression.

**Results:**

Age, gender, tenure, teaching status, and province were not significantly associated with fear of repercussions for nurses or physicians. Mental health nurses had poorer fear responses than their peers outside of these areas, as did community physicians. Strong organization and unit leadership support for safety explained the most variance in fear for both nurses and physicians.

**Conclusions:**

The absence of associations between several plausible factors including age, tenure and teaching status suggests that fear is a complex construct requiring more study. Substantially differing fear responses across locations of care indicate areas where interventions may be needed. In addition, since factors affecting fear of repercussions appear to be different for nurses and physicians, tailoring patient safety initiatives to each group may, in some instances, be fruitful. Although further investigation is needed to examine these and other factors in detail, supportive safety leadership appears to be central to reducing fear of reporting errors for both nurses and physicians.

**Electronic supplementary material:**

The online version of this article (doi:10.1186/s12913-015-0987-9) contains supplementary material, which is available to authorized users.

## Background

Modern health care facilities perform an incredible range of patient services under extreme time pressures and frequent resource shortages. Furthermore, they do this with a complex and diverse chain of staff disciplines interacting to provide care for patients with a wide range of acuity. Under these conditions, health care facilities must abandon their historical expectations of clinician perfection and take up tools beyond punishment to stem the human and financial costs of preventable medical errors [1, 2].

To accomplish this, health care organizations have looked to lessons learned much earlier by other complex industries that also balance productivity and safety, such as aviation, air traffic control, and nuclear power. These high reliability industries manage highly complex technologies and diverse skilled staff, and have learned that when errors occur, punishing staff merely encourages denial, fear, and secrecy [3]. Instead, these industries have moved from a ‘person approach’ that views errors as a result of workers’ personal insufficiency to more of a ‘systems approach’ that accepts humans as fallible and focuses on systemic causes of errors. This latter approach relies on unobstructed reporting of errors to identify and improve work conditions and processes that contribute to errors [4, 5]. However, health care organizations often do not learn from failures, and this failure to learn is, at least in part, due to underreporting of errors and near misses by the frontline clinicians [6]. A number of factors have been identified that explain the low rates of error reporting including the belief that error reporting will not lead to safety improvements [7, 8], confidentiality and legal concerns [9], a punitive work environment [10], traditions of professional autonomy [6], perfectionism [7, 11], power hierarchies within and between professional groups [10, 12, 13], and poorly designed reporting systems [8, 14]. While it is important for health care organizations to take steps to increase the rates of error reporting, they must not lose sight of the fact that error reporting by itself can be counterproductive if it does not lead to error analysis and system improvements [14, 15].

Clinicians cite fear of repercussions as a pivotal obstacle to reporting errors and unsafe practices, though nurses and physicians appear to experience these fears differently. Research suggests that physicians’ reporting of medical errors is restricted chiefly by fear of blame, liability, poor publicity, and estrangement from peers [16, 17]. In contrast, nurses fear disciplinary action from supervisors (e.g., managers or physicians) and feel that errors logged in their files will limit career advancement opportunities [18]. Nurses are more familiar and comfortable with the hospital error reporting process and far outperform physicians or residents in error reporting rates [19, 20]. In contrast, physicians are better at identifying errors and are more likely to disclose errors to patients, though higher rates of disclosure are likely tied to physician-specific regulatory requirements obligating disclosure [21]. On the whole, the literature points to diverse and substantial differences in error reporting behaviours between nurses and physicians, as well as the nature of the repercussions these groups fear. The field of patient safety would benefit from further inquiry in this area.

The present study examines the factors influencing fear of repercussions in nurses and physicians, specifically the role of clinician characteristics (i.e., age, gender, and tenure), organization characteristics (i.e., teaching status, facility size, province, and work area), and safety leadership on nurses’ and physicians’ perceptions of fear of repercussions for reporting and talking about patient safety events. A focus on safety leadership is justified by the fact that, through their actions, priorities, and use of rewards and punishments, leaders can create a safety climate that makes frontline providers feel either safe or afraid to report errors and speak up regarding safety issues [10, 12, 14, 22]. Indeed, this is what makes safety leadership a critical dimension of safety climate. To this end, we emphasize that the concepts of safety climate and safety culture must be carefully distinguished, though they share important interrelationships. Our approach views safety climate as reflecting employees’ perceptions of the extent to which safety is a genuine priority for the unit/department and for the organization. It arises from and provides a window into safety culture, which is comprised of the more deeply held beliefs and values of a setting or organization [23, 24]. For this reason, climate and culture are often theorized as complementary constructs wherein ‘climate can serve as a window through which organizational culture can be viewed’ [24, 25].

For a positive patient safety climate to exist, theoretical [26, 27] as well as empirical investigations [28, 29] argue that strong, credible and visible support for patient safety initiatives by organization leaders is central to a positive patient safety climate. A study of training interventions to enhance patient safety climate perceptions in hospital nurse managers noted that leadership support for safety improvement explained more variance in fear of repercussions for reporting than the safety training intervention itself [30]. Other work noted that leadership support for safety is one of the most important and psychometrically robust dimensions of patient safety climate in the safety literature [31, 32] and is best developed by learning in leadership teams that are multidisciplinary, supportive, respectful, and not governed by hierarchy and status differences [33]. In addition, employees differentiate between the priorities of senior leaders versus unit supervisors, resulting in the emergence of perceptions of two concurrent safety climates [25, 32]. This may be particularly relevant in loosely coupled organizations such as hospitals where unit supervisors can often exercise discretion in implementing and/or prioritizing safety policies initiated by senior management [34]. For example, a hospital might have robust error reporting systems in place as well as executive leadership that prioritizes safety, but if those in frontline supervisory roles such as attending physicians and clinical department heads fail to report errors-in essence normalizing deviance [35, 36]-then other frontline staff may also be less inclined to report errors.

Studies of other outcomes (risk, injury) and in other industries provide an additional basis for considering further explanatory variables to better understand clinicians’ fear of repercussions. Industrial occupations, shorter tenures, younger and older age, and male gender have been associated with higher injury rates [37–39]. But these factors-especially age and tenure-can be highly correlated and the literature is often equivocal: in the health care literature, some work has shown that tenure may both predict [40] and not predict [41] job satisfaction of clinical staff, while the tenure-workplace climate relationship was shown to be statistically significant [40] as well as non-significant [42].

Finally, hospital teaching status, facility size, province, and work area (also termed department, unit, location of care, or care setting) emerge from the health literature as additional factors that may explain variance in clinicians’ fear of repercussions. In Canada, provinces have their own funding, management, and policy environments which may affect clinical practices. Larger hospitals are more likely than smaller ones to have comprehensive patient safety programs, and teaching facilities have training programs that bring contact with new curricula. Quality studies have focused on work areas such as ICU, OR, and labour and delivery units [43, 44] where greater case acuity, specialized focus, and higher status of physicians and nurses may attract greater resources. By contrast, long-term care and mental health settings have been noted for resource scarcity, quality issues, and inadequate staff training [45, 46], underscoring how different departmental / work area contexts may influence clinicians’ fear of repercussions.

## Methods

The data used in this study were taken from a larger set of data collected by Ginsburg and colleagues to examine the psychometric properties of a patient safety climate survey, the Modified Stanford Instrument [47]. Data were collected from ten multi-site health care organizations in three Canadian provinces (Manitoba, Ontario and Nova Scotia) in 2005 and 2006. These organizations provide a full range of clinical services, including acute care, pre-hospital care, long-term care, community care, and mental health services. Human resource departments supplied staff data, including name, job title and unit/department (and site, for multi-site organizations), which allowed all direct care providers (i.e., nurses, physicians, allied health professionals, and technicians), clinical educators, clinical managers, and support service staff with direct patient contact (i.e., unit clerks, housekeeping staff, and health records technicians) to receive a survey. Staff without direct care roles such as those in executive leadership, administrative departments and research were excluded.

Questionnaire administration involved mailing of a personalized cover letter and survey to all eligible staff. A modified Dillman approach [48] was utilized to increase the response rate that involved a reminder card at 2 weeks, followed by a full mailing 3 weeks later to all non-respondents. The cover letter provided detailed information regarding consent to participate and emphasized the voluntary nature of participation, and noted that return of a completed questionnaire constituted consent to participate in the study. Ethics approval was obtained from York University’s Human Participants Review Committee, Capital Health Research Ethics Board and IWK Research Ethics Board (both in Halifax, Nova Scotia), and University Health Network Research Ethics Board (Toronto, Ontario) which required their own approval.

### Study sample

In the fall 2006 dataset collected by Ginsburg and colleagues, 6243 of 22,623 surveys were returned for a response rate of 28 %. Of these responders, 2320 were nurses (30.4 % response rate) and 386 were physicians (23.6 % response rate) [47]. The present study focuses on the nurse and physician respondents from this dataset.

### Instrument

The Modified Stanford Instrument-2006 (MSI-06) consists of 38 items that measure several dimensions of patient safety climate along with basic demographics (e.g., care setting, age, and tenure). The psychometrics of the instrument were reported previously for a broad sample of clinicians and non-clinicians [47]. In the present study, exploratory factor analysis (EFA) was conducted separately for nurses and physicians in order to probe possible difference in dimensionality for these groups (see online supplementary material). We reviewed items loadings on each factor for suitability chiefly by their factor loadings, but also considered theoretical links between items and constructs when considering cross-loading items [49]. Reliability was assessed through Cronbach’s alpha calculations. Together, these analyses identified four non-trivial patient safety climate dimensions reasonably similar in theme and item composition to the original exploratory factor analysis where staff groups were not separated [47]. The factors that emerged from our EFA were used as our outcome variable and two of our explanatory variables.

### Measures

#### Outcome variables

Six items on the MSI-06 were observed to load onto a latent “fear of repercussions” dimension (e.g. “I will suffer negative consequences if I report a patient safety problem”). Reliability was acceptable to good (Cronbach’s α = 0.64 for nurses, 0.70 for physicians) and comparable to the previously reported EFA for non-separated staff groups (α = 0.69, test-retest *r* = 0.64) [47]. Each of the six items were measured using a 5-point agree-disagree Likert-type response scale, and negatively-worded items were reverse coded so that highest score represented most desirable outcome. Provided that a respondent answered at least three of the six items, a mean dimension score was calculated.

#### Explanatory variables

Clinician tenure and gender were obtained from the MSI-06 as an ordinal variable of five levels and a categorical variable of two levels, respectively. Province was obtained from administrative data, as was teaching status (yes/no) of each facility. Location of care was coded from three variables assessing setting and sector obtained from both administrative data and survey data; codes were applied only to cases where these two sources were concordant. Seven locations of interest were identified from the literature and coded as seven dummy variables (emergency department, operating room, critical care unit, long-term care, community, paediatrics, and mental health).

Two further explanatory variables were derived from our EFA. The first was a scale comprised of nine Likert-type items (e.g. “Senior management considers patient safety when program changes are discussed”) loading onto an ‘organization leadership support for safety’ factor, which reflects staff perceptions of the value and priority placed on patient safety by senior leadership in their organization. As done previously, items were reverse-coded where needed and a mean score calculated for respondents answering at least half of the component items. Strong reliability was observed (Cronbach’s α of 0.89 for nurses, 0.90 for physicians), comparable to previously-reported results for un-separated staff groups (α = 0.88, test-retest *r* = 0.82) [47].

Using an identical protocol, the second explanatory variable generated from the EFA was calculated from the four items observed to load onto a ‘unit leadership support for safety’ dimension. These unit-focussed items (e.g. “My supervisor/manager overlooks patient safety problems that happen over and over”) were originally taken from the Agency for Healthcare Research and Quality (AHRQ) supervisory leadership scale [50]. We observed strong reliability (Cronbach’s α = 0.79 for nurses, 0.75 for physicians) which again was comparable to the study by Ginsburg et al. (α = 0.81, test-retest *r* = 0.82) [47].

### Analyses

In addition to the EFA described above, bivariate analyses were performed separately for nurses and physicians to determine unadjusted associations between explanatory and outcome variables (see supplementary material). All explanatory variables except for facility size, which was excessively correlated with teaching status, were carried into the hierarchical linear regression analyses with the fear scale as the outcome variable. We also carried out multiple linear regression analyses where each of the six component items of the fear scale were treated as outcome variables to evaluate if any differences emerged among nurses’ and physicians’ responses to individual fear items. Multicollinearity was not observed between any of the explanatory variables (Pearson *r* < 0.7 and tolerance > 0.10 in all tests).

Separate hierarchical linear regressions were performed for nurses and physicians to identify unique contributions of each explanatory variable (or blocks of explanatory variables) to explaining the variance in fear of repercussions. Demographic variables were entered as the first step in the hierarchical regression analysis as they are static variables of interest and should be entered before the dynamic variables [51], with clinician-level variables (age, gender, and tenure) entered as block 1 and organization-level variables (teaching status, province, and location of care) as block 2.

Organization leadership support for safety and unit leadership support for safety were entered in blocks 3 and 4, respectively, to assess the unique variance in fear of repercussions accounted for by these two explanatory variables. The hierarchical (blocked) regression results were interpreted by examining change in R^2^, reflecting the additional variance explained by each block of variables. The statistical significance was reported at *p* < 0.05.

## Results

Table 1 presents the descriptive statistics of the study sample. Nurse respondents were overwhelmingly female (95 %), while only one-third of physicians were female. About half of both nurses and physicians reported greater than 10 years tenure in their organization, whereas only 5 % had less than 1 year of tenure. Most of the nurses (60 %) and physicians (80 %) worked in teaching facilities. Half of all nurse respondents worked in Nova Scotia, compared with 79 % of physician respondents. Regarding location of care, the largest group of nurses (16 %) worked in long term care settings while most physician respondents worked in the community (20 %). The proportion of physicians (14 %) and nurses (86 %) in our respondent group was similar to their proportions in the full sample (i.e., 18 % physicians and 82 % nurses). We did not have other demographic information for the full sample that would have allowed us to examine further aspects of representativeness.

**Table 1:** Sample Characteristics

	Nurses (*N* = 2319)	Physicians (*N* = 386)
	*n* (%)	*n* (%)
Age (years)		
<30	238 (10.3)	14 (3.6)
31–40	412 (17.8)	82 (21.2)
41–50	727 (31.3)	98 (25.4)
51–60	590 (25.4)	103 (26.7)
>60	224 (9.7)	71 (18.4)
Gender		
Female	2191 (94.5)	134 (34.7)
Male	80 (3.4)	245 (63.5)
Tenure (years)		
<1	119 (5.1)	21 (5.4)
1–2	174 (7.5)	33 (8.5)
3–5	326 (14.1)	69 (17.9)
6–10	290 (12.5)	67 (17.4)
>10	1360 (58.6)	191 (49.5)
Teaching hospital		
Non-teaching	890 (38.4)	81 (21.0)
Teaching	1429 (61.6)	305 (79.0)
Province		
Manitoba	673 (29)	34 (8.8)
Ontario	452 (19.5)	47 (12.2)
Nova Scotia	1186 (51.1)	305 (79.0)
Location of care:		
ED	87 (3.8)	13 (3.4)
OR	106 (4.6)	46 (11.9)
CCU	248 (10.7)	2 (0.5)
LTC	371 (16.0)	21 (5.4)
Community	164 (7.1)	78 (20.2)
Paediatrics	196 (8.5)	52 (13.5)
Mental health	122 (5.3)	23 (6.0)
Other specified areas	1024 (44.2)	149 (38.6)

Totals may not equal 100% due to missing values

Hierarchical regression analyses (Table 2) illustrate that clinician demographic variables (age, gender, and tenure) do not explain a significant amount of variance in fear of repercussions either for nurses (ΔR^2^ = .000, NS) or physicians (ΔR^2^ = .011, NS). Organizational demographic variables (teaching status, province, and location of care) explains a significant amount of additional variance in fear of repercussions for nurses (∆R^2^ = .025, *p* ≤ .001) and physicians (∆R^2^ = .097, *p* ≤ .05). Within location of care, however, it is important to note that only results for paediatrics nurses (β = .130, *p* ≤ .01), mental health nurses (β = −.196, *p* ≤ .001), and community physicians (β = −.436, *p* ≤ .001) are significant.

**Table 2:** Hierarchical linear regression for nurses and physicians. Outcome variable: Fear of Repercussions

	Nurses				Physicians			
	B coefficients in model#:				B coefficients in model#:			
**Block**	**1**	**2**	**3**	**4**	**1**	**2**	**3**	**4**
Block 1: Clinician demographics								
Age	–.006	.006	–.002	–.002	–.008	.003	–.004	.000
Male gender	–.020	–.002	.030	.034	.115	.076	.061	.060
Tenure	.009	.007	.023	.019	–.015	–.002	.020	.022
Block 2: Organization demographics								
Teaching hospital	–	.025	.049	.044	–	–.191	–.110	–.083
Province: ^†^								
Manitoba	–	–.050	–.013	–.006	–	.186	.133	.149
Ontario	–	.040	–.028	–.026	–	.054	–.155	–.134
Location of care								
ER	–	–.067	–.006	.026	–	–.029	–.040	.049
OR	–	.049	–.028	–.031	–	.104	.107	.098
CCU	–	–.019	–.019	–.012	–	–.262	–.525	–.427
LTC	–	–.012	–.085	–.100^b^	–	–.040	–.089	–.050
Community	–	–.088	–.128^b^	–.135^b^	–	–.436^c^	–.405^c^	−355^c^
Paediatrics	–	.130^b^	.024	.030	–	.078	.020	.050
Mental health	–	–.196^c^	–.129^a^	–.118^a^	–	.168	.159	.100
Block 3:								
Organization leadership	–	–	.226^c^	.095^c^	–	–	351^c^	.201^c^
Block 4:								
Unit leadership	–	–	–	.221^c^	–	–	–	.259^c^
Total R^2^	.000	.026^c^	.139^c^	.184^c^	.011	.108^a^	.338^c^	.384^c^
Change in R^2^	.000	.025	.113	.045	.011	.097	.230	.046

^a ^*p* < 0.05

^b ^*p* < 0.01

^c ^*p* < 0.001

^†^ Reference province: Nova Scotia

Organization leadership support for safety (block 3) explains a larger and significant amount of variance in fear of repercussions for nurses (∆R^2^ = .113, *p* ≤ .001) and physicians (∆R^2^ = .230, *p* ≤ .01), over and above variance explained by clinician and organizational demographic variables. Finally, unit leadership support for safety explains a significant amount of additional variance in fear of repercussions for nurses (∆R^2^ = .045, *p* ≤ .001) and physicians (∆R^2^ = .046, *p* ≤ .01) over and above variance explained by the previous blocks of variables. Overall, our explanatory variables explain roughly 18 and 38 % of variance in fear of repercussions for nurses and physicians, respectively.

In addition, the six component items of the fear of repercussions variable were examined individually (see Additional file 1, 2, 3 for multivariate analyses). Among other findings, no age or gender associations were seen, but increased tenure was associated with small but significant improvements in fear scores in nurses for three items, but not for physicians. Location of care, and safety leadership at unit and organization levels displayed numerous associations, largely following the patterns reported for the fear of repercussions described above.

## Discussion

This study examines factors associated with fear of repercussions among physicians and nurses. Clinician level demographics (age, gender, and tenure) were found to have no significant associations with the fear of repercussion scale for either nurses or physicians. This is consistent with mixed results in the literature concerning non-fear outcomes [40–42]. However, in analysis of the six component items of the scale (see Additional file 1, 2, 3), increased organizational tenure was significantly associated with improved scores on three items for nurses but not for physicians, suggesting that nurses may develop confidence around patient safety with time in their organization through processes or conditions that are absent for physicians. Future work could explore this further by collecting time in unit as well as time in organization and probing these separately, thus disaggregating temporal effects of unit-and organization-level patient safety climate processes and leading to a richer exploration of the tenure associations reported in this study.

The influence of location of care was quite mixed. Overall quality issues observed in the literature noted earlier appeared to affect fear perceptions. For instance, nurses working in mental health scored poorly on the fear scale, suggesting that unique aspects of this context such as high nurse turnover, insufficient training and support, and low pay in this work area may be obstacles to an open, unhindered reporting climate. On the other hand, locations expected to be high performers with low fear scores-ED, OR and CCU units, with their high intrinsic risk, specialist staff, and high visibility-did not show more positive fear scores for either nurses or physicians. Community physicians showed fairly poor fear scores, possibly because they conceptualize errors and reporting differently due to their unique clinical context which includes greater distance from the reporting and disciplinary structures of hospital physicians. Additionally, this distance from a central organization may deprive community clinicians of the patient safety training opportunities, peer support, and leadership that is available to their more connected institutional peers. Finally, the non-institutionalized patients comprising community nurses’ and physicians’ practices tend to have greater medical independence and may participate much more in their own care [52, 53]. They may therefore assume much of the responsibility for patient safety that would otherwise remain with a clinician in a hospital setting. Given recent research that supports differences in climate perceptions between nurses and physicians in office-based settings and hospital settings [54], the varying fear scores we found across locations of care warrant further exploration.

Consistent with recent work on factors that influence speaking up about safety issues [55], we found that organization and unit leadership support for safety showed a sweeping and unambiguously positive effect on fear of repercussions, though with different magnitudes of effect for nurses and physicians. Organization leadership explained more than twice the amount of variance in fear of repercussions among physicians than it did in nurses (nurses = 11.3 %, physicians = 23 %) while the effect of unit leadership was smaller and roughly identical for both physicians (4.6 %) and nurses (4.5 %). Some of this variation may arise from differing work structures: nurses work with direct and frequent supervision from their unit, while contact with organizational leadership may be more distant and infrequent. In general, physicians lack close unit/departmental supervision, and may work in several units in one organization, perhaps increasing the relative effect of organization leadership over unit leadership.

Despite the large sample size and diversity of facilities surveyed, a number of limitations to our study exist. Reliability of the fear scale was only moderate, though consistent with previous fear scales established by others using either the same instrument [47] or one sharing many of the same items [30, 56]. This may arise from items being interpreted differently by different disciplines or even work areas. For example, physicians have a different view than nurses of what constitutes a reportable “error” or patient safety problem [19]. In our study, five of the six components of the fear scale ask responders to consider “an error”, “a mistake” or “a patient safety problem”; a nurse may visualize a situation of a very different scope or severity than a physician (recent work has shown these two groups perceive harm quite differently) [57]. While we did examine the scale alpha for nurses and physicians separately to ensure it was reasonable for both groups, this finding speaks to challenges noted by others when selecting one instrument to survey multiple disciplines or work areas [58].

Other limitations included a low response rate, especially for physicians, although these rates were comparable or higher than those reported for similar large studies [34, 56]. Nonetheless, non-response bias may exist. Second, cross-sectional self-report study designs cannot assert causality and can be subject to social desirability biases which may impact levels of fear, but should have little effect on the relationships we examined. Nonetheless, future longitudinal studies could provide valuable insight into how relationships between demographic factors, organizational factors, and fear of repercussions play out over time, just as mixed methods approaches would offer a broader examination of clinician perceptions of safety leadership. Third, common methods bias may inflate relationships given that two explanatory variables reflecting perceptions of organizational and supervisory leadership support for safety were derived from the same source as our outcome variable. Finally, the study data were collected several years ago in 2006; while we anticipate that the relationships we examine are unlikely to be substantially different over time and speaking up about safety issues continues to be an important patient safety area in need of attention [55, 59], we hope that future studies will continue these lines of inquiry with newer data. Indeed, the patient safety climate instrument used in the present study (MSI-06) was recently revised to yield a more parsimonious and psychometrically robust measure of safety climate perceptions-the Canadian Patient Safety Climate Survey (Can-PSCS) [24]. Revised dimensions pertaining to enabling open communication on this instrument along with stronger alphas may strengthen future work in this area. At present, however, since most of the six fear of repercussions items examined here were not carried into the Can-PSCS in order to achieve its parsimony, the MSI-06 offers the most recent suitable data with which to explore our present research questions.

### Study implications

The findings of our study address several knowledge gaps and have implications for both research and practice.

### Implications for research

We report several novel observations regarding fear of repercussions as an outcome variable that may provide a springboard for further inquiry. First, our study found interesting variations in fear perceptions of nurses and physicians across care settings that merit further empirical investigations, especially the poor results among mental health nurses and community care physicians and nurses, and the lack of strong scores in settings where high risk and specialist staffing suggested they should exist (ED, OR and CCU units). In addition, the observed wide-reaching effect of leadership-and differing effects of organization versus unit-level leadership-on fear perceptions of both nurses and physicians emphasizes the importance of leadership support and the need for more inquiry into conditions for strong safety leadership [60]. To that end, recent mixed methods work has offered insights into optimizing learning environments through which diverse groups of managers can learn to work together as leadership teams [33, 61]. Finally, extending our work to smaller hospitals, which were underrepresented in this sample, may be particularly fruitful given recent work showing that the relationship between safety leadership and learning from patient safety failures is significantly stronger in small hospitals [62]. Our results are consistent with recent work on speaking up about patient safety issues which notes the importance of hospital leadership support for speaking up [55] and different predictors of speaking up for physicians and nurses [57], and should be generalizable to the wide variety of jurisdictions with health care organizational structures and safety climate similar to those found in Canada. Research in this area should continue by building on recent work about willingness to speak up among patients [63].

### Implications for practice

The evident differences between nurses’ and physicians’ perceptions of fear of error reporting suggest that no single change is likely to produce universal improvements in patient safety in both professional groups. At the very least, the differing results for nurses and physicians suggest it may be advantageous to tailor at least some ‘speaking up’ improvement strategies for each discipline. However, our observation that increased tenure was associated with several improved item scores in nurses but not physicians is troubling and suggests that supportive processes occurring in nurses over time may not be occurring with physicians. Accordingly, any discipline-specific ‘speaking up’ training should be balanced with learning in inter-professional teams to ensure that beneficial practices developing in one discipline are shared across the care team and to ensure ‘speaking up’ strategies address any barriers that stem from cross-disciplinary authority gradients. Along with training, establishing milestones of patient safety competence via evaluation throughout the career course is valuable, including at baseline during entry to practice [64].

Finally, our results suggest that tailored patient safety strategies may be needed for different care settings (e.g. mental health nurses and community care) to decrease fear of repercussions for reporting and talking about errors and near misses. The multiple divergent results between nurses and physicians may support existing calls for more patient safety learning to be done in inter-professional teams in order to harmonize performance and enhance teamwork. Across settings, our study reaffirms the considerable power of leadership support for safety on positive nurses’ and physicians’ fear perceptions. Health care organizations need to provide leadership training opportunities for senior and supervisory leaders to help them move from bureaucratic to more participative leadership styles so they hear about frontline staff safety concerns and can better integrate solutions in decision-making processes [34]. New views may offer merit too, such as those that look neither solely at individual actors nor organizations but examine where and how individuals are located in hierarchies, and the roles of autonomy, authority, and expertise in those structures [65].

## Conclusions

When clinicians are afraid to report patient safety problems, a key pathway to reducing morbidity and mortality from medical error fails. This study presents early evidence of factors influencing this fear of repercussions in a diverse sample of physicians and nurses. Moreover, we use separate analyses for physicians and nurses in an effort to comprehensively identify differences in fear perceptions between these two disciplines. The diversity of clinician and organizational characteristics in this sample—e.g., age, tenure, locations of care, hospital size-improves the generalizability of these findings. As a whole, our findings indicate a continued predominance of clinical climate based on blame and fear. For patient safety to improve, further empirical investigations as well as tailored safety interventions will be required before these environments give way to a more open and effective reporting climate in health care.

## Additional files

Additional file 1: **Factor loadings for a four factor patient safety climate model for nurses and physicians.** (DOCX 21 kb)
Additional file 2: **Bivariate analyses for fear scale.** (DOCX 21 kb)
Additional file 3: **Multiple linear regression analyses for component items of fear scale.** (DOCX 45 kb)
