# Supplementary material for: Understanding nurses’ and physicians’ fear of repercussions for reporting errors: clinician characteristics, organization demographics, or leadership factors?
Source: BMC Health Serv Res. 2015 Aug 14;15:326. doi: 10.1186/s12913-015-0987-9 (PMC4542128; doi:10.1186/s12913-015-0987-9)
Supplement: Additional file 1: — Factor loadings for a four factor patient safety climate model for nurses and physicians. (DOCX 21 kb) [file 12913_2015_987_MOESM1_ESM.docx]

**Appendix 1:** Factor loadings for a four factor patient safety climate model for nurses and physicians

|  | | Nurses | | | | Physicians | | | |
| --- | --- | --- | --- | --- | --- | --- | --- | --- | --- |
|  |  | Factor no.: | | | | Factor no.: | | | |
|  |  | 1 | 2 | 3 | 4 | 1 | 2 | 3 | 4 |
| Dimension 1: Organization Leadership  for Safety | q1 Patient safety decisions are made at the proper level | .714 | .001 | .063 | -.011 | .734 | .028 | .016 | .015 |
|  | q2 Good communication flow exists up the chain of command | .745 | -.061 | .065 | -.025 | .802 | .082 | -.099 | .069 |
|  | q4 Senior management has a clear picture of the risk | .682 | -.085 | .046 | .046 | .768 | .106 | .003 | -.044 |
|  | q5 My unit takes the time to identify and assess risks | .651 | .008 | -.047 | -.150 | .606 | .063 | -.087 | -.267 |
|  | q6 My unit does a good job managing risks | .715 | .049 | -.003 | -.129 | .552 | -.093 | -.250 | -.206 |
|  | q7 Senior management provides a climate that promotes safety | .759 | -.129 | -.035 | .002 | .808 | -.028 | .062 | -.055 |
|  | q12 Senior management considers patient safety when program changes | .626 | -.179 | -.011 | .017 | .742 | -.021 | .227 | .031 |
|  | q29 My organization balances need for patient safety and productivity | .590 | -.156 | .162 | .065 | .685 | -.157 | .065 | .111 |
|  | q30 I work in an environment where patient safety is a high priority | .596 | -.183 | .036 | -.077 | .787 | .056 | -.028 | -.059 |
| Dimension 2: Unit Leadership for Safety | q33 My supervisor says good word when job done according to safety procedures | .118 | -.789 | -.087 | .126 | .549 | -.202 | .001 | -.040 |
|  | q34 My supervisor seriously considers staff suggestions for improving safety | .273 | -.694 | -.123 | -.044 | .545 | -.298 | .084 | -.125 |
|  | q35 When pressure builds up, supervisor wants us to work faster, even if means shortcuts | -.075 | .580 | -.123 | .198 | -.045 | .746 | -.065 | .142 |
|  | q36 My supervisor overlooks recurring patient safety problems | -.167 | .535 | .019 | .187 | -.135 | .612 | -.174 | .235 |

|  | | Nurses | | | | Physicians | | | |
| --- | --- | --- | --- | --- | --- | --- | --- | --- | --- |
|  |  | Factor no.: | | | | Factor no.: | | | |
|  |  | 1 | 2 | 3 | 4 | 1 | 2 | 3 | 4 |
| Dimension 3: Perceived state of safety | q11 I am less effective when fatigued | .013 | -.012 | -.641 | -.156 | .061 | .149 | .519 | -.044 |
|  | q13 personal problems can adversely affect my performance | .170 | .123 | -.580 | -.037 | .151 | -.014 | .579 | .003 |
|  | q21 loss of experienced personnel has negatively affected my ability to provide high quality patient care | -.217 | .061 | -.444 | .037 | -.026 | .418 | .110 | -.031 |
|  | q22 I have enough time to complete patient care tasks safely | .366 | -.109 | .380 | .038 | .189 | -.372 | -.235 | -.150 |
|  | q24 I witnessed a co-worker do something unsafe to save time | -.183 | -.017 | -.435 | .129 | -.049 | .561 | .112 | .065 |
|  | q25 I am provided with adequate resources to provide safe patient care | .544 | -.116 | .253 | .092 | .537 | -.222 | -.109 | .138 |
|  | q26 I have made significant errors in my work due to my own fatigue | .085 | .077 | -.502 | .236 | -.085 | .014 | .623 | .179 |
|  | q27 I believe that health care error is a real and significant risk to patients we treat | -.113 | -.177 | -.432 | -.050 | .044 | .351 | .377 | -.186 |
|  | q28 I believe health care errors often go unreported | -.103 | -.008 | -.567 | -.037 | -.056 | .299 | .501 | -.096 |
| Dimension 4: Fear of repercussions | q3 Reporting a patient safety problem will result in negative consequences for person reporting | -.370 | .063 | .006 | .514 | -.318 | .160 | -.003 | .475 |
|  | q8 Asking for help is a sign of incompetence | -.139 | .157 | .043 | .536 | -.015 | .222 | -.046 | .491 |
|  | q9 If I make a mistake that has significant consequences and nobody notices, I don't tell anyone about it | .172 | .250 | -.121 | .428 | .017 | -.014 | .188 | .536 |
|  | q16 I will suffer negative consequences if I report a patient safety problem | -.237 | .117 | -.068 | .631 | -.251 | .189 | -.044 | .593 |
|  | q17 If people find out that I made a mistake, I will be disciplined | -.042 | -.066 | -.044 | .584 | .036 | .135 | -.167 | .650 |
|  | q23 Clinicians who make serious mistakes are usually punished | .042 | -.223 | .094 | .580 | .099 | -.101 | -.029 | .627 |
