# Supplementary material for: Understanding nurses’ and physicians’ fear of repercussions for reporting errors: clinician characteristics, organization demographics, or leadership factors?
Source: BMC Health Serv Res. 2015 Aug 14;15:326. doi: 10.1186/s12913-015-0987-9 (PMC4542128; doi:10.1186/s12913-015-0987-9)
Supplement: Additional file 2: — Bivariate analyses for fear scale. (DOCX 21 kb) [file 12913_2015_987_MOESM2_ESM.docx]

**Appendix 2:** Bivariate analyses for fear scale

Statistically significant associations (p<0.05) are indicated in **bold face**.

For all fear outcomes, a higher score indicates more desirable outcome.

|  | Fear scale - Nurses (n=2319**)** | | | | Fear scale - Physicians (n=386**)** | | | | | |
| --- | --- | --- | --- | --- | --- | --- | --- | --- | --- | --- |
|  | n |  | SD | p | n |  | | SD | | p |
| **TOTAL** | 2313 | 3.930 | 0.519 |  | 380 | 3.925 | | 0.535 | |  |
|  |  |  |  |  |  |  | |  | |  |
| Age (yrs) |  |  |  | .785 |  |  | |  | | .586 |
| <30 | 237 | 3.963 | 0.480 |  | 14 | 3.714 | | 0.310 | |  |
| 31-40 | 412 | 3.907 | 0.500 |  | 82 | 3.959 | | 0.461 | |  |
| 41-50 | 726 | 3.927 | 0.529 |  | 97 | 3.975 | | 0.541 | |  |
| 51-60 | 587 | 3.933 | 0.534 |  | 100 | 3.892 | | 0.622 | |  |
| >60 | 224 | 3.937 | 0.502 |  | 69 | 3.921 | | 0.528 | |  |
| Gender |  |  |  | .520 |  |  | |  | | .342 |
| Female | 2186 | 3.934 | 0.515 |  | 133 | 3.890 | | 0.530 | |  |
| Male | 80 | 3.896 | 0.481 |  | 240 | 3.945 | | 0.534 | |  |
| Tenure (yrs) |  |  |  | .305 |  |  | |  | | .728 |
| <1 | 119 | 3.900 | 0.525 |  | 21 | 4.006 | | 0.476 | |  |
| 1-2 | 173 | 3.915 | 0.543 |  | 33 | 3.867 | | 0.518 | |  |
| 3-5 | 325 | 3.935 | 0.484 |  | 69 | 3.909 | | 0.516 | |  |
| 6-10 | 289 | 3.887 | 0.525 |  | 67 | 3.986 | | 0.596 | |  |
| >10 | 1358 | 3.939 | 0.520 |  | 186 | 3.906 | | 0.523 | |  |
| Facility size (beds) |  |  |  | **.000** |  |  | |  | | .270 |
| <100 | 793 | 3.861 | 0.540 | * | 54 | 3.826 | | 0.448 | |  |
| 100-400 | 138 | 3.906 | 0.483 |  | 37 | 3.885 | | 0.593 | |  |
| >400 | 1382 | 3.972 | 0.506 | * | 289 | 3.948 | | 0.542 | |  |
| Teaching hospital |  |  |  | **.000** |  |  | |  | | **.049** |
| Non-teaching | 888 | 3.869 | 0.536 |  | 80 | 3.820 | | 0.481 | |  |
| Teaching | 1425 | 3.968 | 0.505 |  | 300 | 3.953 | | 0.546 | |  |
| Province |  |  |  | **.000** |  |  | |  | | .325 |
| Manitoba | 672 | 3.858 | 0.543 | *,† | 34 | 3.865 | | 0.452 | |  |
| Ontario | 452 | 3.990 | 0.551 | † | 47 | 4.028 | | 0.519 | |  |
| Nova Scotia | 1181 | 3.950 | 0.485 | * | 299 | 3.915 | | 0.546 | |  |
| Location of care |  |  |  |  |  |  |  | |  | |
| ER |  |  |  | .472 |  |  |  | | .086 | |
| yes | 86 | 3.891 | 0.507 |  | 13 | 3.949 | 0.284 | |  | |
| no | 1912 | 3.932 | 0.523 |  | 254 | 3.901 | 0.563 | |  | |
| OR |  |  |  | .178 |  |  |  | | .412 | |
| yes | 106 | 3.997 | 0.507 |  | 46 | 3.964 | 0.612 | |  | |
| no | 1892 | 3.927 | 0.523 |  | 221 | 3.891 | 0.539 | |  | |
| CCU |  |  |  | .653 |  |  |  | | .119 | |
| yes | 248 | 3.944 | 0.503 |  | 2 | 3.583 | 0.118 | |  | |
| no | 1750 | 3.928 | 0.525 |  | 265 | 3.906 | 0.553 | |  | |
| LTC |  |  |  | .112 |  |  |  | | .994 | |
| yes | 371 | 3.889 | 0.541 |  | 21 | 3.921 | 0.529 | |  | |
| no | 1858 | 3.936 | 0.516 |  | 334 | 3.920 | 0.532 | |  | |
| community |  |  |  | **.020** |  |  |  | | **.000** | |
| yes | 163 | 3.822 | 0.610 |  | 75 | 3.688 | 0.549 | |  | |
| no | 2066 | 3.937 | 0.512 |  | 280 | 3.982 | 0.509 | |  | |
| paediatrics |  |  |  | **.000** |  |  |  | | .071 | |
| yes | 196 | 4.055 | 0.442 |  | 52 | 4.050 | 0.536 | |  | |
| no | 2033 | 3.921 | 0.523 |  | 321 | 3.906 | 0.529 | |  | |
| mental h. |  |  |  | **.000** |  |  |  | | .822 | |
| yes | 122 | 3.732 | 0.545 |  | 23 | 3.951 | 0.706 | |  | |
| no | 2107 | 3.945 | 0.514 |  | 350 | 3.925 | 0.520 | |  | |
|  |  |  |  |  |  |  |  | |  | |
|  | n | B | SD | p | n | B | SD | | p | |
| F1 Organizational Leadership | 2310 | .207 | .013 | **.000** | 376 | .257 | .034 | | **.000** | |
| F2 Unit Leadership | 2301 | .288 | .014 | **.000** | 297 | .400 | .042 | | **.000** | |

B = B coefficient (unstandardized)

SE = standard error of the B coefficient

p = significance

* post-hoc tests (Fisher's LSD) are significant between these two levels of the variable

† post-hoc tests (Fisher's LSD) are significant between these two levels of the variable
