# Supplementary material for: Understanding nurses’ and physicians’ fear of repercussions for reporting errors: clinician characteristics, organization demographics, or leadership factors?
Source: BMC Health Serv Res. 2015 Aug 14;15:326. doi: 10.1186/s12913-015-0987-9 (PMC4542128; doi:10.1186/s12913-015-0987-9)
Supplement: Additional file 3: — Multiple linear regression analyses for component items of fear scale. (DOCX 45 kb) [file 12913_2015_987_MOESM3_ESM.docx]

**Appendix 3:** Multiple linear regression analyses for component items of fear scale

Statistically significant associations (p<0.05) are indicated in **bold face**.

For all fear outcomes, a higher score indicates more desirable outcome.

| *q3. Reporting a patient safety problem will result in negative consequences for the person reporting it.* | | | | | | | | | | |
| --- | --- | --- | --- | --- | --- | --- | --- | --- | --- | --- |
|  | Nurses (N=1757) | | | | | Physicians (N=207) | | | | |
|  | B | SE | β | p | tol | B | SE | β | p | tol |
| Age (yrs) | .001 | .019 | .002 | .949 | .773 | -.014 | .058 | -.017 | .809 | .680 |
| Male gender | -.112 | .106 | -.023 | .289 | .973 | .129 | .125 | .062 | .306 | .865 |
| Tenure (yrs) | .063 | .017 | .084 | **.000** | .795 | .058 | .053 | .074 | .278 | .697 |
| Teaching hospital | .058 | .071 | .030 | .417 | .313 | -.152 | .214 | -.057 | .479 | .485 |
| Province:* |  |  |  |  |  |  |  |  |  |  |
| Manitoba | -.021 | .074 | -.010 | .775 | .341 | .159 | .297 | .038 | .594 | .619 |
| Ontario | -.079 | .056 | -.034 | .158 | .755 | -.154 | .201 | -.051 | .444 | .721 |
| Organization leadership | .302 | .034 | .268 | **.000** | .495 | .631 | .100 | .515 | **.000** | .475 |
| Unit  leadership | .313 | .039 | .238 | **.000** | .513 | .162 | .119 | .109 | .176 | .492 |
| Location of care: |  |  |  |  |  |  |  |  |  |  |
| ER | -.068 | .097 | -.015 | .482 | .947 | -.125 | .269 | -.029 | .644 | .816 |
| OR | -.083 | .088 | -.020 | .346 | .951 | .124 | .166 | .047 | .457 | .784 |
| CCU | -.062 | .063 | -.022 | .325 | .876 | -2.163 | .805 | -.155 | **.008** | .957 |
| LTC | -.048 | .061 | -.019 | .426 | .791 | -.268 | .215 | -.076 | .214 | .859 |
| Community | -.123 | .077 | -.036 | .110 | .877 | -.345 | .172 | -.143 | **.046** | .624 |
| Paediatrics | .037 | .071 | .012 | .601 | .843 | .014 | .173 | .005 | .936 | .786 |
| Mental health | -.238 | .084 | -.061 | **.005** | .937 | .370 | .244 | .096 | .130 | .797 |
|  |  |  |  |  |  |  |  |  |  |  |
| R Square | .232 |  |  |  |  | .392 |  |  |  |  |
| Adjusted R Square | .225 |  |  |  |  | .344 |  |  |  |  |
| Standard Error | .805 |  |  |  |  | .786 |  |  |  |  |

| *q8. Asking for help is a sign of incompetence.* | | | | | | | | | | | | | | | | | | | | | | | | | | | | | | | | | | | | | | | |  |  |
| --- | --- | --- | --- | --- | --- | --- | --- | --- | --- | --- | --- | --- | --- | --- | --- | --- | --- | --- | --- | --- | --- | --- | --- | --- | --- | --- | --- | --- | --- | --- | --- | --- | --- | --- | --- | --- | --- | --- | --- | --- | --- |
|  | | Nurses (N=1753) | | | | | | | | | | | | | | | Physicians (N=208) | | | | | | | | | | | | | | | | | | | | | | |  |  |
|  | | B | SE | | | β | | | p | | | | tol | | | | B | | | | SE | | | | β | | | | | p | | | | | tol | | | | |  |  |
| Age (yrs) | | -.027 | .019 | | | -.036 | | | .161 | | | | .769 | | | | .026 | | | | .056 | | | | .038 | | | | | .641 | | | | | .676 | | | | |  |  |
| Male gender | | .177 | .107 | | | .038 | | | .097 | | | | .971 | | | | -.006 | | | | .121 | | | | -.004 | | | | | .959 | | | | | .863 | | | | |  |  |
| Tenure (yrs) | | -.018 | .018 | | | -.025 | | | .317 | | | | .792 | | | | -.023 | | | | .051 | | | | -.036 | | | | | .654 | | | | | .694 | | | | |  |  |
| Teaching hospital | | .050 | .072 | | | .028 | | | .484 | | | | .315 | | | | -.052 | | | | .206 | | | | -.024 | | | | | .801 | | | | | .485 | | | | |  |  |
| Province:* | |  |  | | |  | | |  | | | |  | | | |  | | | |  | | | |  | | | | |  | | | | |  | | | | |  |  |
| Manitoba | | -.034 | .074 | | | -.018 | | | .646 | | | | .344 | | | | .178 | | | | .285 | | | | .053 | | | | | .533 | | | | | .619 | | | | |  |  |
| Ontario | | -.096 | .056 | | | -.044 | | | .088 | | | | .754 | | | | -.047 | | | | .193 | | | | -.019 | | | | | .809 | | | | | .722 | | | | |  |  |
| Organization leadership | | .100 | .034 | | | .095 | | | **.003** | | | | .495 | | | | .063 | | | | .097 | | | | .063 | | | | | .515 | | | | | .473 | | | | |  |  |
| Unit  leadership | | .321 | .039 | | | .261 | | | **.000** | | | | .514 | | | | .288 | | | | .115 | | | | .240 | | | | | **.013** | | | | | .491 | | | | |  |  |
| Location of care: | |  |  | | |  | | |  | | | |  | | | |  | | | |  | | | |  | | | | |  | | | | |  | | | | |  |  |
| ER | | .119 | .098 | | | .028 | | | .225 | | | | .949 | | | | -.101 | | | | .259 | | | | -.029 | | | | | .698 | | | | | .817 | | | | |  |  |
| OR | | -.138 | .089 | | | -.036 | | | .120 | | | | .952 | | | | .110 | | | | .159 | | | | .052 | | | | | .491 | | | | | .788 | | | | |  |  |
| CCU | | -.008 | .063 | | | -.003 | | | .902 | | | | .875 | | | | .372 | | | | .773 | | | | .033 | | | | | .631 | | | | | .957 | | | | |  |  |
| LTC | | -.092 | .061 | | | -.038 | | | .132 | | | | .793 | | | | .272 | | | | .206 | | | | .095 | | | | | .188 | | | | | .860 | | | | |  |  |
| Community | | -.194 | .077 | | | -.061 | | | **.011** | | | | .875 | | | | -.138 | | | | .165 | | | | -.071 | | | | | .404 | | | | | .626 | | | | |  |  |
| Paediatrics | | -.075 | .072 | | | -.026 | | | .295 | | | | .844 | | | | .145 | | | | .166 | | | | .066 | | | | | .383 | | | | | .787 | | | | |  |  |
| Mental health | | -.074 | .085 | | | -.020 | | | .383 | | | | .937 | | | | -.401 | | | | .234 | | | | -.128 | | | | | .088 | | | | | .797 | | | | |  |  |
|  | |  |  | | |  | | |  | | | |  | | | |  | | | |  | | | |  | | | | |  | | | | |  | | | | |  |  |
| R Square | | .118 |  | | |  | | |  | | | |  | | | | .143 | | | |  | | | |  | | | | |  | | | | |  | | | | |  |  |
| Adjusted R Square | | .110 |  | | |  | | |  | | | |  | | | | .076 | | | |  | | | |  | | | | |  | | | | |  | | | | |  |  |
| Standard Error | | .809 |  | | |  | | |  | | | |  | | | | .755 | | | |  | | | |  | | | | |  | | | | |  | | | | |  |  |
| *q9. If I make a mistake that has significant consequences and nobody notices, I do not tell anyone about it.* | | | | | | | | | | | | | | | | | | | | | | | | | | | | | | | | | | | | | | |  |  |  |
|  | | Nurses (N=1747) | | | | | | | | | | | | | | | | Physicians (N=208) | | | | | | | | | | | | | | | | | | | | |  |  |  |
|  | | B | | | SE | | | Β | | p | | | | tol | | | | B | | | | SE | | | | β | | | | | p | | | | | tol | | |  |  |  |
| Age (yrs) | | -.003 | | | .017 | | | -.004 | | .867 | | | | .772 | | | | .001 | | | | .049 | | | | .002 | | | | | .984 | | | | | .676 | | |  |  |  |
| Male gender | | .001 | | | .093 | | | .000 | | .992 | | | | .972 | | | | -.058 | | | | .106 | | | | -.038 | | | | | .585 | | | | | .863 | | |  |  |  |
| Tenure (yrs) | | -.028 | | | .015 | | | -.047 | | .074 | | | | .794 | | | | -.023 | | | | .045 | | | | -.039 | | | | | .616 | | | | | .694 | | |  |  |  |
| Teaching hospital | | .056 | | | .063 | | | .038 | | .371 | | | | .315 | | | | -.043 | | | | .181 | | | | -.022 | | | | | .814 | | | | | .485 | | |  |  |  |
| Province:* | |  | | |  | | |  | |  | | | |  | | | |  | | | |  | | | |  | | | | |  | | | | |  | | |  |  |  |
| Manitoba | | .062 | | | .065 | | | .038 | | .341 | | | | .345 | | | | .274 | | | | .251 | | | | .091 | | | | | .277 | | | | | .619 | | |  |  |  |
| Ontario | | -.092 | | | .049 | | | -.051 | | .060 | | | | .757 | | | | .098 | | | | .170 | | | | .044 | | | | | .564 | | | | | .722 | | |  |  |  |
| Organization leadership | | -.002 | | | .030 | | | -.002 | | .943 | | | | .494 | | | | -.145 | | | | .085 | | | | -.162 | | | | | .089 | | | | | .473 | | |  |  |  |
| Unit  leadership | | .189 | | | .034 | | | .183 | | **.000** | | | | .512 | | | | .340 | | | | .101 | | | | .314 | | | | | **.001** | | | | | .491 | | |  |  |  |
| Location of care: | |  | | |  | | |  | |  | | | |  | | | |  | | | |  | | | |  | | | | |  | | | | |  | | |  |  |  |
| ER | | .114 | | | .086 | | | .032 | | .182 | | | | .948 | | | | -.069 | | | | .228 | | | | -.022 | | | | | .763 | | | | | .817 | | |  |  |  |
| OR | | .023 | | | .077 | | | .007 | | .766 | | | | .951 | | | | .085 | | | | .140 | | | | .045 | | | | | .545 | | | | | .788 | | |  |  |  |
| CCU | | -.065 | | | .055 | | | -.030 | | .240 | | | | .875 | | | | -1.490 | | | | .681 | | | | -.146 | | | | | **.030** | | | | | .957 | | |  |  |  |
| LTC | | -.104 | | | .053 | | | -.051 | | .052 | | | | .790 | | | | -.119 | | | | .181 | | | | -.046 | | | | | .511 | | | | | .860 | | |  |  |  |
| Community | | -.138 | | | .067 | | | -.052 | | **.040** | | | | .874 | | | | -.446 | | | | .145 | | | | -.254 | | | | | **.002** | | | | | .626 | | |  |  |  |
| Paediatrics | | .030 | | | .063 | | | .012 | | .635 | | | | .843 | | | | .205 | | | | .146 | | | | .103 | | | | | .162 | | | | | .787 | | |  |  |  |
| Mental health | | .036 | | | .074 | | | .012 | | .624 | | | | .938 | | | | -.017 | | | | .206 | | | | -.006 | | | | | .934 | | | | | .797 | | |  |  |  |
|  | |  | | |  | | |  | |  | | | |  | | | |  | | | |  | | | |  | | | | |  | | | | |  | | |  |  |  |
| R Square | | .040 | | |  | | |  | |  | | | |  | | | | .180 | | | |  | | | |  | | | | |  | | | | |  | | |  |  |  |
| Adjusted R Square | | .032 | | |  | | |  | |  | | | |  | | | | .116 | | | |  | | | |  | | | | |  | | | | |  | | |  |  |  |
| Standard Error | | .707 | | |  | | |  | |  | | | |  | | | | .665 | | | |  | | | |  | | | | |  | | | | |  | | |  |  |  |
| *q16. I will suffer negative consequences if I report a patient safety problem.* | | | | | | | | | | | | | | | | | | | | | | | | | | | | | | | | | | | | | | | |  |  |
|  | Nurses (N=1757) | | | | | | | | | | | | | | | | | | Physicians (N=206) | | | | | | | | | | | | | | | | | | | | |  |  |
|  | B | | | SE | | | β | | | | p | | | | tol | | | | B | | | | SE | | | | β | | | | | p | | | | | tol | | |  |  |
| Age (yrs) | -.002 | | | .017 | | | -.003 | | | | .888 | | | | .773 | | | | -.038 | | | | .056 | | | | -.047 | | | | | .498 | | | | | .686 | | |  |  |
| Male gender | -.074 | | | .096 | | | -.017 | | | | .442 | | | | .972 | | | | .051 | | | | .120 | | | | .026 | | | | | .672 | | | | | .866 | | |  |  |
| Tenure (yrs) | .043 | | | .016 | | | .065 | | | | **.007** | | | | .794 | | | | .060 | | | | .051 | | | | .080 | | | | | .245 | | | | | .696 | | |  |  |
| Teaching hospital | .091 | | | .065 | | | .054 | | | | .160 | | | | .313 | | | | .157 | | | | .206 | | | | .063 | | | | | .446 | | | | | .479 | | |  |  |
| Province:* |  | | |  | | |  | | | |  | | | |  | | | |  | | | |  | | | |  | | | | |  | | | | |  | | |  |  |
| Manitoba | -.007 | | | .068 | | | -.004 | | | | .912 | | | | .342 | | | | .455 | | | | .284 | | | | .117 | | | | | .111 | | | | | .619 | | |  |  |
| Ontario | -.084 | | | .051 | | | -.041 | | | | .099 | | | | .754 | | | | -.321 | | | | .192 | | | | -.113 | | | | | .097 | | | | | .720 | | |  |  |
| Organization leadership | .219 | | | .031 | | | .219 | | | | **.000** | | | | .494 | | | | .452 | | | | .096 | | | | .392 | | | | | **.000** | | | | | .473 | | |  |  |
| Unit  leadership | .289 | | | .035 | | | .248 | | | | **.000** | | | | .512 | | | | .285 | | | | .114 | | | | .204 | | | | | **.014** | | | | | .490 | | |  |  |
| Location of care: |  | | |  | | |  | | | |  | | | |  | | | |  | | | |  | | | |  | | | | |  | | | | |  | | |  |  |
| ER | .112 | | | .089 | | | .028 | | | | .208 | | | | .948 | | | | .392 | | | | .258 | | | | .096 | | | | | .130 | | | | | .816 | | |  |  |
| OR | -.113 | | | .080 | | | -.031 | | | | .156 | | | | .952 | | | | .466 | | | | .159 | | | | .190 | | | | | **.004** | | | | | .789 | | |  |  |
| CCU | -.075 | | | .057 | | | -.030 | | | | .187 | | | | .877 | | | | -.273 | | | | .769 | | | | -.021 | | | | | .723 | | | | | .957 | | |  |  |
| LTC | -.081 | | | .055 | | | -.036 | | | | .142 | | | | .793 | | | | -.122 | | | | .205 | | | | -.037 | | | | | .551 | | | | | .860 | | |  |  |
| Community | -.073 | | | .070 | | | -.024 | | | | .297 | | | | .874 | | | | -.323 | | | | .167 | | | | -.140 | | | | | .055 | | | | | .625 | | |  |  |
| Paediatrics | -.081 | | | .065 | | | -.029 | | | | .212 | | | | .842 | | | | .005 | | | | .165 | | | | .002 | | | | | .976 | | | | | .785 | | |  |  |
| Mental health | -.197 | | | .077 | | | -.057 | | | | **.010** | | | | .936 | | | | .400 | | | | .239 | | | | .107 | | | | | .096 | | | | | .809 | | |  |  |
|  |  | | |  | | |  | | | |  | | | |  | | | |  | | | |  | | | |  | | | | |  | | | | |  | | |  |  |
| R Square | .190 | | |  | | |  | | | |  | | | |  | | | | .377 | | | |  | | | |  | | | | |  | | | | |  | | |  |  |
| Adjusted R Square | .183 | | |  | | |  | | | |  | | | |  | | | | .327 | | | |  | | | |  | | | | |  | | | | |  | | |  |  |
| Standard Error | .733 | | |  | | |  | | | |  | | | |  | | | | .751 | | | |  | | | |  | | | | |  | | | | |  | | |  |  |
| *q17. If people find out that I made a mistake, I will be disciplined.* | | | | | | | | | | | | | | | | | | | | | | | | | | | | | | | | | | | | | | | | |  |
|  | Nurses (N=1738) | | | | | | | | | | | | | | | | | | | Physicians (N=207) | | | | | | | | | | | | | | | | | | | | |  |
|  | B | | | | SE | | | β | | | | P | | | | tol | | | | B | | | | SE | | | | β | | | | | p | | | | | tol | | |  |
| Age (yrs) | .013 | | | | .022 | | | .015 | | | | .569 | | | | .770 | | | | -.056 | | | | .065 | | | | -.070 | | | | | .386 | | | | | .672 | | |  |
| Male gender | .155 | | | | .123 | | | .030 | | | | .210 | | | | .973 | | | | .072 | | | | .139 | | | | .037 | | | | | .604 | | | | | .865 | | |  |
| Tenure (yrs) | .010 | | | | .021 | | | .013 | | | | .633 | | | | .792 | | | | .073 | | | | .060 | | | | .096 | | | | | .228 | | | | | .688 | | |  |
| Teaching hospital | -.051 | | | | .083 | | | -.026 | | | | .540 | | | | .313 | | | | -.276 | | | | .237 | | | | -.110 | | | | | .245 | | | | | .485 | | |  |
| Province:* |  | | | |  | | |  | | | |  | | | |  | | | |  | | | |  | | | |  | | | | |  | | | | |  | | |  |
| Manitoba | -.081 | | | | .086 | | | -.038 | | | | .350 | | | | .342 | | | | .209 | | | | .329 | | | | .053 | | | | | .526 | | | | | .619 | | |  |
| Ontario | .047 | | | | .065 | | | .020 | | | | .469 | | | | .754 | | | | -.018 | | | | .222 | | | | -.006 | | | | | .936 | | | | | .720 | | |  |
| Organization leadership | .016 | | | | .039 | | | .014 | | | | .682 | | | | .499 | | | | .203 | | | | .111 | | | | .174 | | | | | .070 | | | | | .477 | | |  |
| Unit  leadership | .172 | | | | .045 | | | .127 | | | | **.000** | | | | .517 | | | | .247 | | | | .133 | | | | .174 | | | | | .064 | | | | | .497 | | |  |
| Location of care: |  | | | |  | | |  | | | |  | | | |  | | | |  | | | |  | | | |  | | | | |  | | | | |  | | |  |
| ER | -.238 | | | | .113 | | | -.051 | | | | **.036** | | | | .947 | | | | .133 | | | | .298 | | | | .033 | | | | | .655 | | | | | .817 | | |  |
| OR | -.011 | | | | .103 | | | -.003 | | | | .912 | | | | .951 | | | | -.024 | | | | .186 | | | | -.010 | | | | | .896 | | | | | .791 | | |  |
| CCU | .012 | | | | .073 | | | .004 | | | | .871 | | | | .874 | | | | .304 | | | | .890 | | | | .023 | | | | | .733 | | | | | .957 | | |  |
| LTC | -.154 | | | | .071 | | | -.058 | | | | **.029** | | | | .792 | | | | -.173 | | | | .237 | | | | -.052 | | | | | .466 | | | | | .860 | | |  |
| Community | -.221 | | | | .090 | | | -.062 | | | | **.014** | | | | .875 | | | | -.514 | | | | .190 | | | | -.226 | | | | | **.007** | | | | | .626 | | |  |
| Paediatrics | .170 | | | | .083 | | | .052 | | | | **.042** | | | | .842 | | | | -.077 | | | | .191 | | | | -.030 | | | | | .687 | | | | | .787 | | |  |
| Mental health | -.081 | | | | .099 | | | -.020 | | | | .413 | | | | .934 | | | | .087 | | | | .269 | | | | .024 | | | | | .747 | | | | | .797 | | |  |
|  |  | | | |  | | |  | | | |  | | | |  | | | |  | | | |  | | | |  | | | | |  | | | | |  | | |  |
| R Square | .034 | | | |  | | |  | | | |  | | | |  | | | | .171 | | | |  | | | |  | | | | |  | | | | |  | | |  |
| Adjusted R Square | .026 | | | |  | | |  | | | |  | | | |  | | | | .106 | | | |  | | | |  | | | | |  | | | | |  | | |  |
| Standard Error | .933 | | | |  | | |  | | | |  | | | |  | | | | .869 | | | |  | | | |  | | | | |  | | | | |  | | |  |
| *q23. Clinicians who make serious mistakes are usually punished.* | | | | | | | | | | | | | | | | | | | | | | | | | | | | | | | | | | | | | | | | | |
|  | | Nurses (N=1680) | | | | | | | | | | | | | | | | | | Physicians (N=205) | | | | | | | | | | | | | | | | | | | | | |
|  | | B | | | SE | | | β | | | | p | | | | tol | | | | B | | | | SE | | | | | Β | | | | | p | | | | tol | | | |
| Age (yrs) | | -.006 | | | .021 | | | -.009 | | | | .755 | | | | .770 | | | | .063 | | | | .067 | | | | | .080 | | | | | .347 | | | | .672 | | | |
| Male gender | | .052 | | | .113 | | | .011 | | | | .648 | | | | .976 | | | | .187 | | | | .145 | | | | | .097 | | | | | .199 | | | | .864 | | | |
| Tenure (yrs) | | .051 | | | .019 | | | .073 | | | | **.008** | | | | .791 | | | | .010 | | | | .062 | | | | | .013 | | | | | .878 | | | | .696 | | | |
| Teaching hospital | | .053 | | | .077 | | | .030 | | | | .489 | | | | .317 | | | | -.033 | | | | .254 | | | | | -.013 | | | | | .895 | | | | .468 | | | |
| Province:* | |  | | |  | | |  | | | |  | | | |  | | | |  | | | |  | | | | |  | | | | |  | | | |  | | | |
| Manitoba | | .031 | | | .080 | | | .016 | | | | .694 | | | | .348 | | | | -.270 | | | | .347 | | | | | -.070 | | | | | .438 | | | | .598 | | | |
| Ontario | | .142 | | | .060 | | | .065 | | | | **.019** | | | | .756 | | | | -.402 | | | | .235 | | | | | -.141 | | | | | .088 | | | | .724 | | | |
| Organization leadership | | -.087 | | | .036 | | | -.082 | | | | **.017** | | | | .495 | | | | -.015 | | | | .116 | | | | | -.013 | | | | | .899 | | | | .482 | | | |
| Unit  leadership | | .012 | | | .042 | | | .010 | | | | .777 | | | | .512 | | | | .227 | | | | .137 | | | | | .163 | | | | | .100 | | | | .502 | | | |
| Location of care: | |  | | |  | | |  | | | |  | | | |  | | | |  | | | |  | | | | |  | | | | |  | | | |  | | | |
| ER | | .120 | | | .103 | | | .029 | | | | .243 | | | | .947 | | | | .136 | | | | .311 | | | | | .034 | | | | | .664 | | | | .807 | | | |
| OR | | .127 | | | .094 | | | .033 | | | | .180 | | | | .950 | | | | -.153 | | | | .191 | | | | | -.063 | | | | | .426 | | | | .783 | | | |
| CCU | | .143 | | | .067 | | | .055 | | | | **.032** | | | | .874 | | | | .713 | | | | .924 | | | | | .055 | | | | | .442 | | | | .957 | | | |
| LTC | | -.113 | | | .066 | | | -.047 | | | | .085 | | | | .789 | | | | .161 | | | | .247 | | | | | .049 | | | | | .516 | | | | .854 | | | |
| Community | | -.160 | | | .083 | | | -.049 | | | | .055 | | | | .873 | | | | -.301 | | | | .199 | | | | | -.135 | | | | | .133 | | | | .613 | | | |
| Paediatrics | | .148 | | | .076 | | | .051 | | | | .051 | | | | .839 | | | | .039 | | | | .199 | | | | | .016 | | | | | .843 | | | | .783 | | | |
| Mental health | | -.092 | | | .090 | | | -.025 | | | | .306 | | | | .939 | | | | .165 | | | | .280 | | | | | .046 | | | | | .557 | | | | .796 | | | |
|  | |  | | |  | | |  | | | |  | | | |  | | | |  | | | |  | | | | |  | | | | |  | | | |  | | | |
| R Square | | .033 | | |  | | |  | | | |  | | | |  | | | | .078 | | | |  | | | | |  | | | | |  | | | |  | | | |
| Adjusted R Square | | .024 | | |  | | |  | | | |  | | | |  | | | | .005 | | | |  | | | | |  | | | | |  | | | |  | | | |
| Standard Error | | .849 | | |  | | |  | | | |  | | | |  | | | | .902 | | | |  | | | | |  | | | | |  | | | |  | | | |

* Reference province: Nova Scotia
